# Protective effect of melatonin on soluble Aβ_1–42_-induced memory impairment, astrogliosis, and synaptic dysfunction via the Musashi1/Notch1/Hes1 signaling pathway in the rat hippocampus

**DOI:** 10.1186/s13195-016-0206-x

**Published:** 2016-09-15

**Authors:** Shuman Zhang, Pan Wang, Lili Ren, Chunli Hu, Jing Bi

**Affiliations:** Department of Neurobiology and Key Laboratory of Neurodegenerative Diseases of Liaoning Province, Jinzhou Medical University, No. 40, Section 3, Songpo Road, Linghe District, Jinzhou, Liaoning 121000 China

**Keywords:** Melatonin, Soluble Aβ_1–42_, Notch1, Hes1, Musashi1, Learning and memory, Neuroplasticity

## Abstract

**Background:**

Amyloid-beta (Aβ) plays a key role in Alzheimer’s disease (AD) pathogenesis, and soluble Aβ oligomers are more cytotoxic than Aβ fibrils. Recent evidence suggests that Notch signaling is affected by AD and other brain diseases. Melatonin exerts beneficial effects on many aspects of AD and may protect against myocardial ischemia via Notch1 signaling regulation. Therefore, we hypothesized that the Notch1 signaling pathway is involved in the neuroprotective role of melatonin against soluble Aβ_1–42_.

**Methods:**

An AD rat model was established via repeated intracerebroventricular administration of soluble Aβ_1–42_. Melatonin treatment was administered 24 hours prior to Aβ_1–42_ administration via an intraperitoneal injection. The effects of melatonin on spatial learning and memory, synaptic plasticity, and astrogliosis were investigated. The expression of several Notch1 signaling components, including Notch1, the Notch1 intracellular domain (NICD), Hairy and enhancer of split 1 (Hes1, a downstream effector of Notch), and Musashi1 (a positive regulator of Notch), were examined using immunohistochemistry, western blotting, and quantitative real-time PCR. In vitro studies were conducted to determine whether the melatonin-mediated protection against Aβ_1–42_ was inhibited by DAPT, an inhibitor of Notch signaling.

**Results:**

Melatonin improved the Aβ_1–42_-induced impairment in spatial learning and memory, attenuated synaptic dysfunction, and reduced astrogliosis. Melatonin also ameliorated the effects of Aβ_1–42_ on Notch1, NICD, Hes1, and Musashi1. The in vitro studies demonstrated that DAPT effectively blocked the neuroprotective effect of melatonin against Aβ_1–42_.

**Conclusions:**

These findings suggest that melatonin may improve the soluble Aβ_1–42_-induced impairment of spatial learning and memory, synaptic plasticity, and astrogliosis via the Musashi1/Notch1/Hes1 signaling pathway.

## Background

Alzheimer’s disease (AD) is the most prevalent cause of dementia in older individuals, and it comprises an irreversible and progressive neurodegenerative disorder. AD is characterized by the formation of extracellular amyloid plaques and intracellular neurofibrillary tangles [1]. The amyloid-beta (Aβ) peptide comprises the main component of amyloid plaques. Aβ_1–42_ accumulation in the brain plays a crucial role in AD pathogenesis and has been proposed as a trigger for AD onset and progression [2, 3]. Over the previous 15 years, substantial evidence has suggested that soluble Aβ oligomers (AβOs) play pivotal roles in the synaptic dysfunction, neurodegeneration, neuroinflammation, and cognitive deficits present in AD [4, 5]. Moreover, AβOs are attractive targets for therapeutics and diagnostics because of their early and unifying pathological appearance [5–7].

Notch signaling is an evolutionarily conserved pathway that plays an important role in central nervous system development. Recent studies have demonstrated that the Notch1 signaling pathway regulates neurogenesis, neuronal networks, synaptic plasticity, and learning and memory in adult brains [8, 9]. The central component of Notch1 signaling is the Notch1 receptor protein, a single-pass transmembrane protein that maintains an inactivated state without ligand binding [10]. Following ligand binding, the Notch1 receptor is activated and the Notch1 intracellular domain (NICD) is released into the cytosol. The NICD subsequently translocates to the nucleus and regulates its target *genes*, such as *Hairy and enhancer of split* (*HES*) [11]. *HES* belongs to the basic helix–loop–helix (bHLH) family of transcription factors and plays regulatory roles in neuronal function and morphology [11]. *Hes1* is one of seven members of the *HES* gene family and is activated by notch signaling. In addition, the neural RNA-binding protein Musashi1 plays a key role in the activation of Notch1 signaling by inhibiting the translation of the Notch1 inhibitor Numb [12]. Recently, the Notch1 signaling pathway has been demonstrated to participate in aging processes and multiple age-related neurodegenerative diseases, such as AD [8, 9]. Notch signaling is reduced in the aged brain, and Notch dysfunction is closely related to learning and memory deficits [9]. Furthermore, an important role for Notch signaling in AD has recently been identified. Studies have reported that the Notch signaling activity was decreased in familial AD (FAD) mutations of Presenilin1, whereas other studies have demonstrated that Notch signaling was activated in sporadic AD [8]. Thus, the exact functional role of the Notch signaling pathway in the onset and progression of AD remains unclear. Musashi1 comprises a key regulator of Notch1 signaling, and its downstream protein Numb is important for AD pathogenesis [13]. However, the extent to which Musashi1 and Notch signaling participate in the mechanisms that underlie AD pathogenesis is unknown.

Melatonin is a lipophilic neurohormone that has numerous physiological roles, such as a regulator of circadian rhythms, a protector of mitochondria, an antioxidant and anti-inflammatory agent, and a neuroprotectant [14–18]. The beneficial effect of melatonin on neurodegenerative diseases has been widely investigated. In AD patients, the melatonin level was significantly decreased in the serum and cerebrospinal fluid (CSF), and could thus serve as an early diagnostic marker [19–21]. Melatonin supplementation may attenuate Aβ accumulation, neurodegeneration, inflammation, and memory impairment in AD animal models and patients. Therefore, melatonin and its agonist are considered promising therapeutic agents for AD treatment [22, 23]. Accumulating data indicate that melatonin exerts its neuroprotective role in AD through the regulation of several signaling pathways, such as PI3/Akt/GSk3β and hemo-oxygenase-1 [24, 25]. Nevertheless, knowledge regarding the cellular mechanisms of the neuroprotective effect of melatonin in AD is lacking. Additional studies are necessary to elucidate the beneficial actions of melatonin as an AD treatment and to develop pharmaceutical strategies for AD patients. Recently, Yu and colleagues [26] demonstrated that melatonin protected against myocardial ischemia–reperfusion injury via the modulation of the Notch1/Hes1 signaling pathway. Therefore, we hypothesize that melatonin exerts its neuroprotective effect in a soluble Aβ_1–42_ oligomer-induced AD animal model through the Musashi1/Notch1/Hes1 signaling pathway.

In the current study, we initially determined the protective effect of melatonin against soluble Aβ_1–42_ oligomer-induced neurotoxicity and astrogliosis in the hippocampus. We subsequently investigated the regulatory role of melatonin on Notch1, Hes1, and Musashi1. Moreover, we determined whether Notch1 pathway inhibition abolished the neuroprotective role of melatonin against soluble Aβ_1–42_ oligomers by decreasing NICD, Hes1, and Musashi1 expression in vitro.

## Methods

### Animals

Adult male Sprague–Dawley (SD) rats that weighed 250–350 g were purchased from Vital River Laboratory Animal Technology Company (Beijing, China). The rats were acclimated to the laboratory environment for 1 week prior to use. Timed pregnant SD rats were bred in the animal facility at Liaoning Medical University, and the day of vaginal plug detection was designated as embryonic day 0.5 (E0.5). All experimental procedures that involved animals were approved by the Animal Ethics Committee of Jinzhou Medical University and were conducted in accordance with the guidelines of the Animal Care and Use Committee of Jinzhou Medical University.

### Aβ_1–42_ intracerebroventricular administration and melatonin intraperitoneal injection

Soluble Aβ_1–42_ oligomers were prepared according to previously described methods [27, 28]. Briefly, the human Aβ_1–42_ peptide (A9810; Sigma-Aldrich, St. Louis, MO, USA) was dissolved in sterile double-distilled water (DDW) at a concentration of 200 μmol/L and incubated at 37 °C for 24 hours. The preparation was centrifuged at 14,000 × *g* at 4 °C for 10 minutes. The supernatant that contained soluble AβOs was collected and stored at 4 °C. The soluble AβOs were used within 24 hours of preparation.

Melatonin was dissolved in 100 % ethanol to prepare a 0.1 M stock solution and was subsequently diluted to 25 μM with phosphate-buffered saline (PBS).

The detailed protocol for the implantation of guide cannulas and Aβ administration in the left lateral ventricle of the brain has been described previously. The rats were placed in a stereotaxic apparatus (model 51600; Stoelting, Wood Dale, IL, USA) following deep anesthetization with 10 % chloral hydrate (300 mg/kg, intraperitoneal (i.p.) injection). The incised area was disinfected with 75 % ethanol, and a hole was drilled through the left side of the skull. A microdialysis guide cannula (CMA12 Elite microdialysis probe; CMA Microdialysis AB, Solna, Sweden) was implanted in the left cerebral lateral ventricle (coordinates from Bregma: AP = –0.7 mm, ML = +1.7 mm, and DV = −4.0 mm) according to the atlas by Paxinos and Franklin (2000) [29]. The cannula was secured to the skull using screws and dental cement. Three days after the operation, the rats were randomly divided into four groups (*n* = 10–14/group): control (Ctrl, vehicle injection), Aβ_1–42_ (Aβ, intracerebroventricular (i.c.v.) injection), melatonin (Mel, i.p. injection), and Aβ_1–42_ plus melatonin (Aβ + Mel, i.c.v. and i.p. injections).

For the melatonin-treated groups, the rats received 500 mg/kg body weight of melatonin (in 25 % ethanol) via i.p. injection for 16 continuous days between 13:30 and 16:00 hours. The 25 % ethanol solution was used as the control. For the Aβ_1–42_ injection groups, awake and freely moving rats were injected with 80 μmol/L of Aβ_1–42_, freshly diluted with DDW, in a 5 μl volume 1 day after the melatonin injection. Aβ_1–42_ was injected every other day for a total of eight injections using a Hamilton microsyringe (Hamilton, GR, Switzerland) at an infusion rate of 1 μl/min for 5 minutes. Following the injection, the injection needle was maintained in place for an additional 2–3 minutes to prevent reflux. The same volume of DDW was used as the control.

**Fig. 1:**
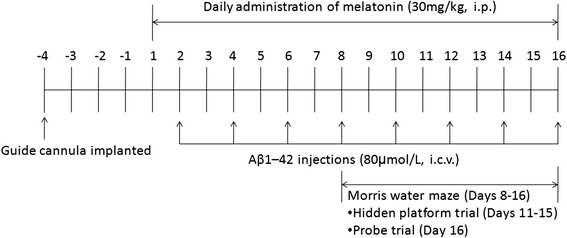
Schematic illustrating the timeline for drug administration and completion of the MWM. *i.p.* intraperitoneal injection, *i.c.v.* intracerebroventricular administration

The detailed timeline of the Aβ_1–42_ i.c.v. administration, melatonin i.p. injections, and completion of the Morris Water Maze (MWM) test is schematically depicted in Fig. 1.

### Morris Water Maze test

Beginning on day 8 of melatonin and Aβ_1–42_ administration, the rats (*n* = 10–14/group) were tested in the MWM as described previously with several modifications (Fig. 1) [28, 30]. Briefly, the test was performed in a circular water pool (diameter 120 cm and height 40 cm) over a 9-day period. The rats underwent four trials per day with a 10-minute intertrial interval. The circular pool was filled with water to a depth of 25 cm, and the temperature was maintained at 24 ± 2 °C. The maze was divided into four quadrants: northeast (NE), northwest (NE), southeast (SW), and southwest (SW). A visible submerged platform (5 cm^2^) was placed in the NE quadrant for days 1–3, and a submerged platform was placed in the SW quadrant for days 4–8 with spatial cues located in the room. The platform was removed on day 9 for the probe trial. Data were collected and analyzed using a video tracking system (ANY-maze Video Tracking Software; Stoelting Co. Wood Dale, IL, USA). The rats were habituated 1 day prior to training with a 120-second swim session in the pool without a platform.

For the first 3 days, the rats were trained to find the visible platform. The platform was placed in the NE quadrant, and a visible sign was located opposite the platform quadrant. In each trial, all rats were given 120 seconds to find the platform. If the rats found the platform, they were allowed to sit on it for 15 seconds. The rats that failed to find the platform within 120 seconds were guided to the platform and allowed to sit on it for 15 seconds.

For acquisition testing, the rats were tested for 5 consecutive days with a hidden platform. The platform was placed in the SW quadrant and submerged 2 cm below the water surface. The other procedures were the same as the procedures used in the first 3 days. Performance was measured by the escape latency, path efficiency, swim speed, and trace for each session.

For probe testing, the platform was removed from the pool on day 9, and the rats were allowed 120 seconds to search for the absent platform prior to removal from the water. Their performances were recorded and analyzed for the time spent in the target quadrant (where the platform was located during the hidden platform training), the number of entries into the target quadrant, and the swim trace.

### Primary neuronal cultures

Primary hippocampal neuron cultures were prepared from day 18 timed pregnant SD rats as described previously with modifications [31]. Briefly, the pregnant rats were deeply anesthetized, and the embryos were removed from the uterus. The hippocampi were dissected and placed in ice-cold Hank’s balanced salt solution (HBSS; Invitrogen). After being cut into small pieces, the hippocampal tissue was digested with 0.25 % trypsin–ethylenediaminetetraacetic acid (EDTA) for 5 minutes at 37 °C. The digestion was terminated by the addition of Minimum Essential Media (MEM; Invitrogen) that contained 10 % fetal bovine serum (FBS). The hippocampal tissue was then mechanically triturated into a single-cell suspension in MEM with a fire-polished sterile Pasteur pipette and centrifuged at 1,000 × *g* for 5 minutes at room temperature (RT). The neurons were resuspended in MEM supplemented with 5 % FBS, 5 % horse serum (HS), 2 % B27, 2 mM glutamine, and 1 % penicillin/streptomycin (P/S; Invitrogen). The neuronal suspensions were subsequently plated on poly-d-lysine-coated 24-well plates with circular glass coverslips or 60 mm tissue culture dishes at a density of 3 × 10^4^ cells/cm^2^. The cultures were maintained at 37 °C in a humidified 5 % CO_2_ incubator (Heratherm 240i; Thermo Scientific, USA). To inhibit the proliferation of glial cells, 2 μM cytosine arabinoside (AraC) was added to the cultures after 2 days. To assess the changes in astrocytes, the primary neurons were cultured without AraC. The neurons were fed every 3 days by removing half of the old media and adding the same volume of fresh culture media. The cultures were used for experiments on culture day 5.

### Cell viability assay (MTT)

For dose-dependent experiments, hippocampal neurons were treated with soluble Aβ_1–42_ at concentrations of 0, 25, 50, 100, or 500 nM for 24 hours or with melatonin at concentrations of 0, 5, 10, 50, or 100 μM for 26 hours. Concentrations of 100 nM for Aβ_1–42_ and 50 μM for melatonin were selected for the other in-vitro experiments. To verify the neuroprotective effect of melatonin against Aβ_1–42_ at the selected dosages, neurons were exposed to vehicle, Aβ_1–42_, melatonin, or Aβ_1–42_ plus melatonin under the same conditions used in the dose-dependent experiments. The cell viability assay was conducted using an MTT kit (ST316; Beyotime Biotechnology, China) according to the manufacturer’s instructions.

### Drug treatment for hippocampal neurons

On day 5, the primary neurons were divided into six groups: a control group (Ctrl) treated with vehicle; an Aβ_1–42_ group (Aβ) treated with Aβ_1–42_ for 24 hours at a final concentration of 100 nM; a melatonin group (Mel) treated with melatonin for 26 hours at a final concentration of 50 μM; an Aβ_1–42_ plus melatonin group (Aβ + Mel) treated with 100 nM Aβ_1–42_ and 50 μM melatonin; a Aβ_1–42_ plus melatonin and DAPT group (Aβ + Mel + D) with a final DAPT (a γ-secretase inhibitor) concentration of 5 μM; and a DAPT group (DAPT) with a final concentration of 5 μM.

### Western blot analysis

Following deep anesthetization, the animals were rapidly decapitated and the brains were removed from the skull. The hippocampi were dissected and processed for protein extraction immediately or after storage at –80 °C. For the primary hippocampal neurons, the cells were rinsed with PBS after treatment and collected for protein extraction. The hippocampal tissue and primary neurons from various groups were homogenized in radio-immunoprecipitation assay (RIPA) buffer (P0013B; Beyotime, China) using sonication. The samples were cleared using centrifugation for 5 minutes at 4 °C. The protein concentration was determined using a bicinchoninic acid (BCA) protein assay kit (Bio-Rad, Hercules, CA, USA). An equal amount of total protein (30 μg) from each sample was separated by SDS-PAGE and subsequently transferred to a polyvinylidene difluoride (PVDF) membrane (Millipore). The membranes were blocked in a blocking solution that contained 0.1 M TBST (Tris-buffered saline with Tween 20) and 0.1 % bovine serum albumin (BSA) for 2 hours at RT. The membranes were subsequently incubated with different primary antibodies diluted in the same blocking solution overnight at 4 °C. The following antibodies were used: glial fibrillary acidic protein (GFAP) (1:2000, 556327; BD), full-length Notch1 (1:1000, sc-6015; Santa Cruz Biotechnology), NICD (1:1000, val1744; Cell Signaling Technology), Hes1 (1:1000, D6P2U; Cell Signaling Technology), and Musashi1 (1:1000, ab52865; Abcam). After three washes with 0.1 % TBST, the membranes were incubated with a horseradish peroxidase (HRP)-conjugated anti-rabbit secondary antibody or anti-mouse IgG (1:2000; Jackson lmmunoResearch Laboratories) at RT for 2 hours. Immunoreactive bands were detected using an Enhanced Chemiluminescence Plus kit (32132; Thermo Scientific, USA). The images were captured with a UVP BioSpectrum Imaging System (UVP, Upland, CA, USA). All membranes were reprobed with a β-actin antibody (1:2000, A2066; Sigma) as an internal control. The relative intensity of each protein was measured using VisionWorks LS analysis software (UVP, LLC, Upland, CA, USA). The percent change in the protein intensity was calculated relative to the vehicle control included in each experiment.

### Immunohistochemistry

Rats were transcardially perfused with 4 % paraformaldehyde (PFA) in 0.1  M PBS (pH 7.4) under deep anesthesia. The brains were dissected and post-fixed in 4 % PFA for 48 hours at 4 °C. After fixation, the brains were equilibrated in 25 % sucrose in PBS for 48 hours at 4 °C and subsequently cut into 30 μm-thick coronal sections using a vibratome (Leica VT1200S; Leica Microsystems, Germany). The sections were stored in PBS with 0.01 % NaN_3_ at 4 °C until further use.

The brain sections were mounted on glass slides and dried at RT. For antigen retrieval, the sections were incubated in 0.01 M citric acid with a pH of 6.0 at 100 °C for 15 minutes and subsequently washed with TBS (PBS containing 0.1 % Triton-X100) three times for 5 minutes per wash. For primary neurons, the cells were rinsed with PBS and fixed with 4 % PFA in PBS for 30 minutes at 4 °C and subsequently washed three times with PBS for 5 minutes per wash. The tissue sections or cells were blocked in 3 % normal donkey serum (NDS) with 0.3 % TritonX-100 in TBST for 60 minutes at RT and subsequently incubated in primary antibody overnight in 3 % NDS and 0.3 % Tween-20 in TBS at 4 °C. The following primary antibodies were used: GFAP (1:2000, 556327; BD), SNAP25 (1:2000, 111011; Synaptic System, Germany), Synaptophysin (1:2000, 611880; BD Biosciences), Notch1 (1:1000, sc-6015; Santa Cruz Biotechnology), NICD (1:1000, ab8925; Abcam), Hes1 (1:1000, D6P2U; Cell Signaling), Musashi1 (1:1000, ab52865; Abcam), βIII-tubulin (1:1000, ab18207; Abcam), and MAP2 (1:800, NB600-1372; Novus biologicals). After rinsing, the tissue sections were incubated with a biotinylated donkey-anti-mouse secondary antibody or rabbit IgG in 1.5 % NDS for 60 minutes at RT and subsequently immersed in 0.3 % H_2_O_2_ for 30 minutes at RT to block endogenous peroxidases. After three washes with TBS, the sections were incubated with solutions from the Vectastain Elite ABC kit (Vector Laboratories) according to the manufacturer’s instructions. The signals were visualized using a TSA Plus Fluorescence Kit (Cyanine 3 or Fluorescein kit; PerkinElmer, Waltham, MA, USA). For primary neurons, the cells were incubated with a donkey-anti-mouse secondary antibody or rabbit Cyanine 3-conjugated IgG for 2 hours at RT after washing with TBS. The tissue sections and cells were counterstained with 4′,6-diamidino-2-phenylindole (DAPI) (1:2000; Invitrogen) and mounted with coverslips using fluorescent mounting medium (Dako, Carpinteria, CA, USA). Images were captured with a fluorescence microscope (DMI4000B; Leica Microsystems, Wetzlar, Germany). In the control experiments, the primary antibody was omitted. All images were obtained under identical settings and analyzed using ImageJ software (NIH).

### RNA isolation and quantitative real-time PCR analysis

Total RNA was isolated from freshly dissected hippocampal tissue or primary hippocampal neurons using TRIzol reagent (Invitrogen) according to the manufacturer’s instructions. The RNA quantity and quality were calculated using A260/A280 readings from a NanoDrop (2000C; Thermo Scientific). cDNA was synthesized using 5 μg of total RNA and the SuperScript® III First-Strand Synthesis System (Invitrogen) with random hexamer primers. Quantitative real-time PCR (qPCR) was performed using an Applied Biosystems 7500 Fast Real-Time PCR System (Applied Biosystems, Carlsbad, CA, USA) and MicroAmp Fast 96-well reaction plates sealed with MicroAmp optical adhesive film. The amplification reactions were performed with SYBR-Green Master Mix (Invitrogen) in a 25 μl reaction volume. All reactions were performed at 50 °C for 2 minutes and 95 °C for 2 minutes followed by 40 cycles of 95 °C for 15 seconds and 60 °C for 30 seconds. The data were analyzed with 7500 Fast Software v2.3. All reactions were performed in triplicate with three animals per group. The glyceraldehyde 3-phosphate dehydrogenase (GAPDH) gene expression levels were used as an internal control. The relative gene expression was calculated via the 2^–ΔΔCt^ method. The following primers were used: Notch1, forward 5′-CGC ACA AGG TGT CTT CCA G-3′ and reverse 5′-AGT AGT TGA GTG TGC GGC-3′ (143 bp); Hes1, forward 5′-TGG AAT AGC GCT ACC GAT CAC-3′ and reverse 5′-GTT GAC TGG GGT AG-3′ (243 bp); and GAPDH, forward 5′-CTA CCC ACG GCA AGT TCA AC-3′ and reverse 5′-CCA GTA GAC TCC ACG ACA TAC-3′.

### Golgi-Cox staining and spine density analysis

Golgi-Cox staining was performed using the FD Rapid GolgiStain Kit (FD Neurotechnologies, Ellicott City, MD, USA) according to the manufacturer’s protocol. Following deep anesthetization, the rats were decapitated and the brains were removed from the skull. The brains were cut into three coronal blocks, immersed in an impregnation solution for 2 weeks at RT, and subsequently transferred to solution C for 48 hours at RT in the dark. They were cut into 100 μm-thick sections using a vibratome (Leica VT1200S; Leica Microsystems, Germany). The sections were mounted on gelatin-coated slides with solution C. After drying at RT, the sections were immerged in a mixture that contained one part solution D, one part solution E, and two parts Milli-Q water for 10 minutes. After washing with Milli-Q water, the sections were dehydrated in graded alcohol solutions, cleared in xylene, and coverslipped. Images for neurons in the dentate gyrus (DG) and CA1 and CA3 regions were obtained with an automated upright microscope (DM6000 B; Leica). The number of spines on the primary branches of basal dendrites were counted using Neurolucida software 10 (MBF Bioscience, Williston, VT, USA).

For the quantitative analysis, three independent sections from each animal were randomly selected. After the images were obtained, 10 neurons from each rat for a total of three rats per group were analyzed. To determine the spine density, the spines on three basal dendrites from a single neuron were counted and the average number of spines per 10 μm segment was calculated.

### Statistical analysis

All experiments were independently repeated at least three times. The data are expressed as the mean ± standard deviation. The statistical analysis was conducted with SPSS 16.0 software (IBM, Somers, NY, USA) using a one-way analysis of variance (ANOVA) followed by Bonferroni post-hoc tests. For the escape latency and swim path efficiency in the MWM test, the data were analyzed using a two-way repeated-measures ANOVA followed by LSD post-hoc tests for multiple group comparisons. The statistical significance level was set to *p* < 0.05.

## Results

**Fig. 2:**
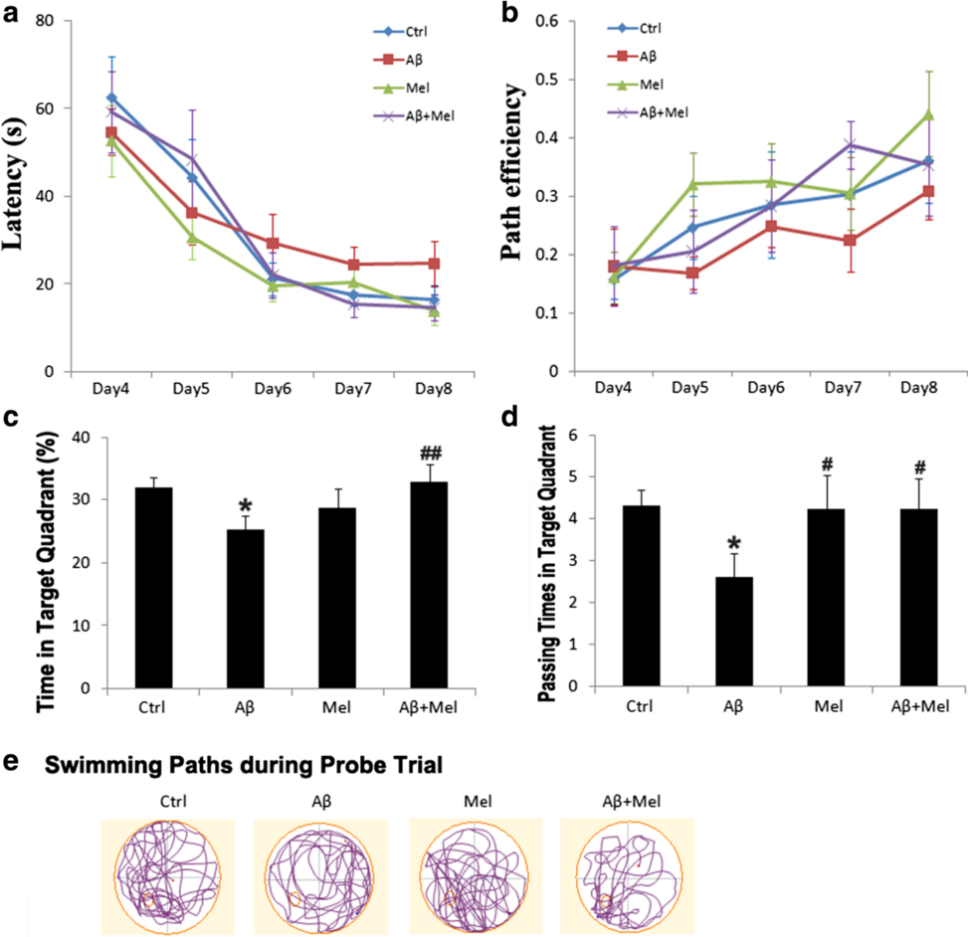
Melatonin improved spatial learning and reference memory in soluble Aβ_1–42_-injected rats. **a** Average escape latency for rats searching for the hidden platform over 5 consecutive training days. **b** Average swimming path efficiency of rats searching for the hidden platform over 5 consecutive training days. **c** Average swimming time of rats in the target quadrant during the probe trial. **d** Average passing times of rats in the target quadrant during the probe trial. **e** Swimming paths of rats during the probe trial. *Large red circle*, water maze pool; *small red circle*, platform location. *s* second, *Ctrl* control group, *Aβ* Aβ_1–42_-injected group, *Mel* melatonin-injected group, *Aβ + Mel* dual Aβ_1–42_ and melatonin-injected group. ^*^ *p* < 0.05 compared with control rats; ^#^ *p* < 0.05 and ^##^ *p* < 0.01 compared with Aβ_1–42_-injected rats

We initially investigated whether melatonin prevented the spatial learning and memory impairments induced by repeated soluble Aβ_1–42_ i.c.v. injections using the MWM test. To assess the spatial learning ability, the rats had to find a hidden platform during 5 consecutive days of acquisition training. The results of a two-way repeated ANOVA indicated that the time to find the platform decreased over the 5 consecutive training days (*F*_(4, 40)_ = 68.622, *p* < 0.001). The main effect of treatment on the escape latency was not significant (*F*_(3, 30)_ = 2.919, *p* = 0.050); however, the results indicated that the Aβ group tended to take longer to reach the platform on days 6–8 compared with the control rats. Furthermore, the Aβ + Mel group tended to take less time to reach the platform on days 6–8 compared with the Aβ group (Fig. 2a). The path efficiency significantly increased over the 5 consecutive training days (*F*_(4, 40)_ = 15.186, *p* < 0.001). There was a significant effect of treatment on the path efficiency (*F*_(3, 30)_ = 3.062, *p* < 0.05). The swimming path of the Aβ group tended to be less efficient on days 6–8 compared with the control group. The swimming path of the Aβ + Mel group tended to be more efficient on days 6–8 compared with the Aβ group (Fig. 2a, b). These findings suggest that the melatonin treatment prevented the soluble Aβ_1–42_-induced impairment in spatial learning.

After acquisition training, the platform was removed and reference memory was assessed using a probe trial. The Aβ_1–42_ i.c.v.-injected rats spent less time in the target quadrant and completed fewer platform crossings compared with the control rats. However, the Aβ_1–42_ rats treated with melatonin spent more time in the target quadrant and completed more platform crossings compared with the Aβ_1–42-_injected rats (Fig. 2c, d). The Aβ_1–42_-injected rats swam randomly with little or no preference for the previous platform location compared with the control rats. However, the Aβ_1–42_ rats treated with melatonin swam more in the previous platform location compared with the Aβ_1–42_-injected rats (Fig. 2e). These findings indicate that pretreatment with melatonin prevented the soluble Aβ_1–42_-induced reference memory impairment.

The swim speeds during the acquisition training and probe trial were not significantly different among the treatment groups (data not shown), which suggests the differences in spatial learning and reference memory were not the result of differences in the swim speed. During the visible platform trials on days 1–3, there were no differences in the latency to reach the platform among the groups (data not shown).

**Fig. 3:**
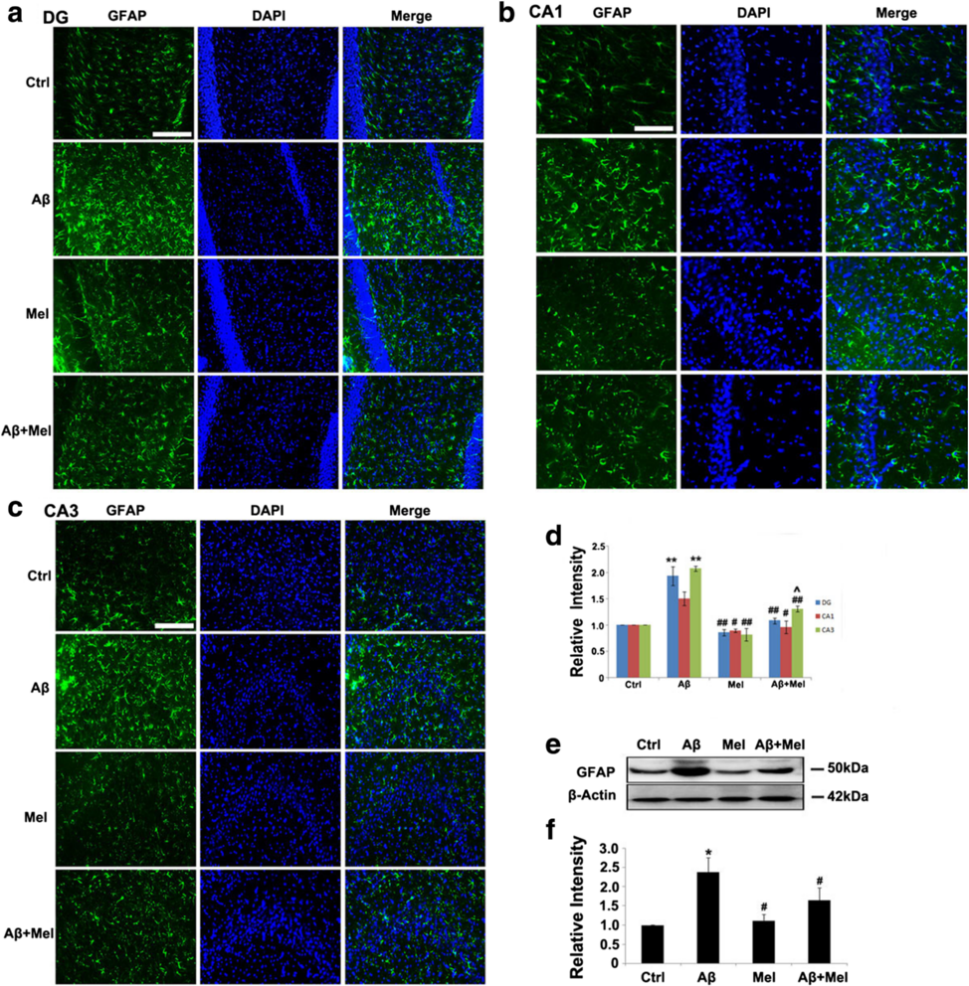
Melatonin reduced astrogliosis in soluble Aβ_1–42_-injected rats. **a** Representative images of immunofluorescent staining for GFAP in the dentate gyrus (*DG*) of the hippocampus. **b** Representative images of immunofluorescent staining for GFAP in the CA1 region of the hippocampus. **c** Representative images of immunofluorescent staining for GFAP in the CA3 region of the hippocampus. **d** Statistical analysis of immunofluorescent staining for GFAP in the DG and the CA1 and CA3 regions. **e** Representative western blot images for GFAP in the hippocampus. **f** Statistical analysis of the relative intensity of GFAP immunoreactive bands from hippocampal tissue samples. *Scale bar* = 100 μm. *p < 0.05 and **p < 0.01 compared with sham-operated rats; ^#^ *p* < 0.05 and ^##^ *p* < 0.01 compared with Aβ_1–42_-injected rats; ^^^ *p* < 0.05 compared with melatonin-injected rats. *DAPI* 4′,6-diamidino-2-phenylindole, *GFAP* glial fibrillary acidic protein, *Ctrl* control group, *Aβ* Aβ_1–42_-injected group, *Mel* melatonin-injected group, *Aβ + Mel* dual Aβ_1–42_ and melatonin-injected group

To investigate the role of melatonin treatment on Aβ_1–42_-induced astrogliosis, we examined the expression of GFAP, a marker of reactive astrocytes, using immunohistochemistry. The GFAP expression in the Aβ_1–42_-injected rats was significantly increased in the DG and CA1 region compared with the control group (Fig. 3a, c, d). The GFAP expression was also increased in the CA1 region of the Aβ group compared with the control group; however, the difference was not significant (Fig. 3b, d). The GFAP expression in all three hippocampal regions of the Aβ + Mel group was significantly decreased compared with the Aβ group, and there were no significant differences between the Aβ + Mel and control groups (Fig. 3a–d). For the melatonin groups, the GFAP expression in all three hippocampal regions was not significantly different compared with the control group (Fig. 3a–d). To quantitatively verify the protective effect of melatonin on Aβ_1–42_-induced astrocyte activation, we performed a western blot analysis of hippocampal tissue. The results confirmed that the GFAP expression was significantly increased in the Aβ_1–42_-injected group compared with the control group and was significantly decreased in the Aβ + Mel group compared with the Aβ group (Fig. 3e, f). The GFAP expression levels were similar between the rats injected with melatonin alone and the Aβ + Mel rats compared with the control group (Fig. 3e, f).

**Fig. 4:**
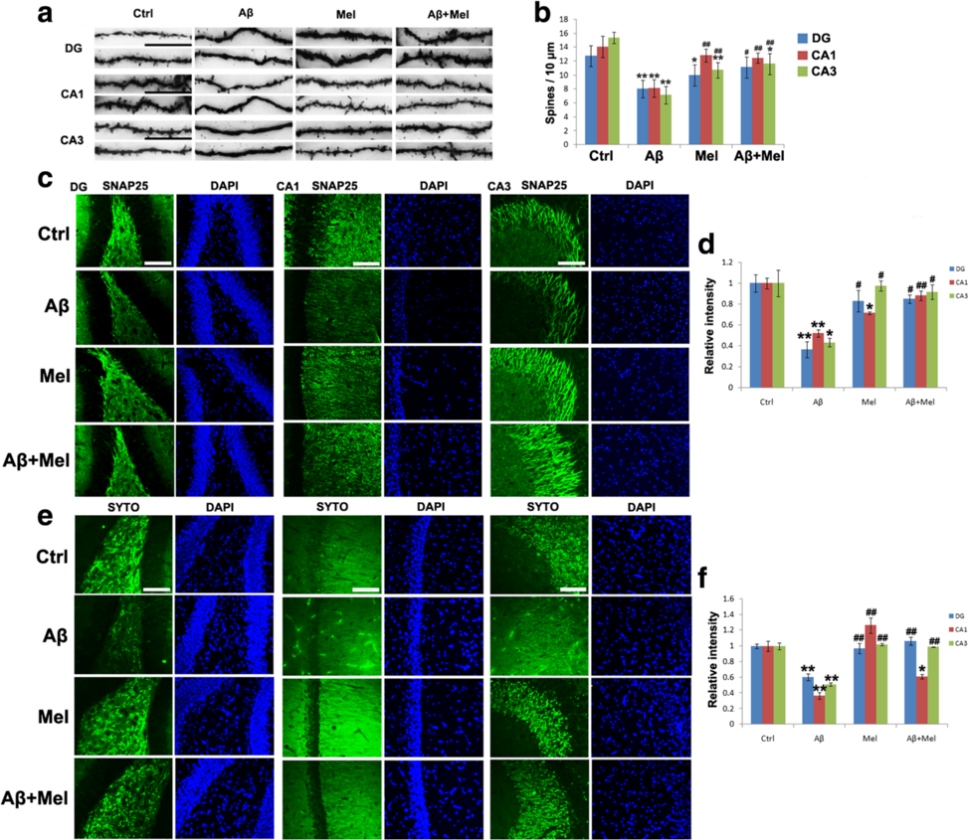
Melatonin restored the dendritic spine number and synaptic density in Aβ_1–42_-injected rats. **a** Representative images of Golgi-Cox staining of dendritic spines in the dentate gyrus (*DG*) and the CA1 and CA3 regions of the hippocampus. **b** Statistical analysis of the average number of Golgi-Cox stained dendritic spines in the DG and the CA1 and CA3 regions. **c** Representative images of immunofluorescent staining for SNAP25 in the DG and the CA1 and CA3 regions of the hippocampus. **d** Statistical analysis of immunofluorescent staining for SNAP25. **e** Representative images of immunofluorescent staining for synaptophysin in the DG and the CA1 and CA3 regions of the hippocampus. **f** Statistical analysis of immunofluorescent staining for synaptophysin. *Scale bar* = 100 μm. *p < 0.05 and **p < 0.01 compared with sham-operated rats; ^#^ *p* < 0.05 and ^##^ *p* < 0.01 compared with Aβ_1–42_-injected rats. *Ctrl* control group, *Aβ* Aβ_1–42_-injected group, *Mel* melatonin-injected group, *Aβ + Mel* dual Aβ_1–42_ and melatonin-injected group, *SYTO* synaptophysin, *DAPI* 4′,6-diamidino-2-phenylindole

We determined whether melatonin protected against Aβ_1–42_-induced synaptotoxicity in the hippocampus under our experimental conditions. Golgi-Cox staining indicated that the spine density of basal dendrites was substantially reduced in the neurons of the DG, CA1, and CA3 regions of the Aβ group compared with the control group (Fig. 4a, b). The spine density was significantly increased in all three hippocampal regions of the Aβ + Mel group compared with the Aβ group; however, the spine density in the CA3 region did not reach the same level identified in the control group (Fig. 4a, b). In the melatonin groups, the spine density was decreased in the DG and the CA3 region compared with the control group; however, there was no significant difference in the CA1 region compared with the control group (Fig. 4a, b). To verify these findings, we examined the expression of the presynaptic proteins SNAP25 and synaptophysin using immunohistochemistry. The results were consistent with the Golgi-Cox staining data. The expression levels of SNAP25 and synaptophysin were significantly decreased in the DG and the CA1 and CA3 regions of the Aβ group compared with the control group (Fig. 4c–f). The reduction in SNAP25 expression induced by Aβ was completely blocked by melatonin treatment in all three hippocampal areas of the Aβ + Mel group, and the reduction in synaptophysin expression induced by Aβ was completely abolished by melatonin treatment in the DG and the CA3 region of the Aβ + Mel group (Fig. 4c–f). Melatonin treatment alone did not affect the expression of SNAP25 or synaptophysin in the three hippocampal regions compared with the control group, with the exception of SNAP25 expression in the CA1 region (Fig. 4c–f). These findings suggest that melatonin protected against the soluble Aβ_1–42_-induced synaptotoxic effect in the hippocampus under our experimental conditions.

**Fig. 5:**
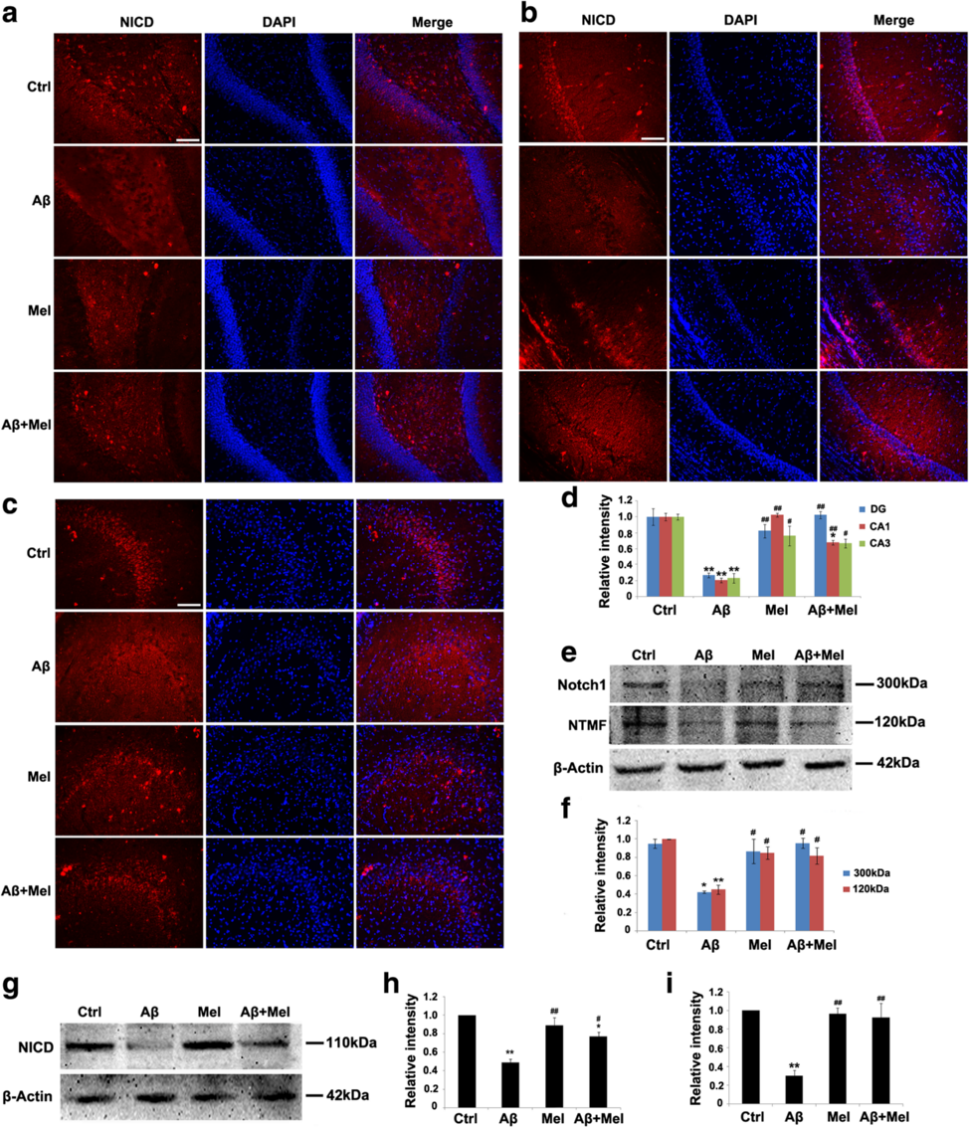
Melatonin upregulated the expression of Notch1 and Notch1 intracellular domain (*NICD*) in soluble Aβ_1–42_-injected rats. **a** Representative images of immunofluorescent staining for NICD in the dentate gyrus (*DG*) of the hippocampus. **b** Representative images of immunofluorescent staining for NICD in the CA1 region of the hippocampus. **c** Representative images of immunofluorescent staining for NICD in the CA3 region of the hippocampus. **d** Statistical analysis of immunofluorescent staining for NICD. **e** Representative western blot images for Notch1 in the hippocampus. **f** Statistical analysis of the relative intensity of Notch1 immunoreactive bands. **g** Representative western blot images for NICD in the hippocampus. **h** Statistical analysis of the relative intensity of NICD immunoreactive bands. **i** *Notch1* gene expression in the hippocampus by qPCR. *Scale bar* = 100 μm. *p < 0.05 and **p < 0.01 compared with sham-operated rats; ^#^ *p* < 0.05 and ^##^ *p* < 0.01 compared with Aβ_1–42_-injected rats. *Ctrl* control group, *Aβ* Aβ_1–42_-injected group, *Mel* melatonin-injected group, *Aβ + Mel* dual Aβ_1–42_ and melatonin-injected group, *DAPI* 4′,6-diamidino-2-phenylindole, *NTMF* Notch1 transmembrane fragment

To determine whether the Notch1 signaling pathway participated in the protective effect of melatonin against Aβ-induced astrogliosis and synaptotoxicity in the hippocampus, we examined the NICD expression using immunohistochemistry. The expression of NICD was significantly decreased in the hippocampal DG and the CA1 and CA3 regions of the Aβ_1–42_ rats compared with the control rats (Fig. 5a–d). However, the decrease in the NICD expression was completely blocked in the DG and the CA3 region and partially blocked in the CA1 region of the Aβ + Mel rats compared with the control and Aβ groups (Fig. 5a–d). There were no obvious differences in the NICD expression in the DG or the CA1 or CA3 region of the Mel group compared with the control group (Fig. 5a–d). To quantitatively analyze the Notch1 and NICD expression, western blot analysis was performed with anti-Notch1 and anti-NICD antibodies. The expression levels of full-length Notch1 (Notch1), the Notch1 transmembrane fragment (NTMF), and NICD were significantly decreased in the Aβ_1–42_-injected rats compared with the control rats (Fig. 5e–h). However, the decrease in the Notch1 and NTMF expression was prevented completely and the decrease in the NICD expression was prevented partially in the Aβ + Mel treatment group compared with the control and Aβ groups (Fig. 5e–h). Melatonin treatment alone did not significantly affect the expression of Notch1, NTF, or NICD in the hippocampus compared with the control group (Fig. 5e–h). *Notch1* gene expression was examined using qPCR. The results were consistent with the western blotting data (Fig. 5i). These findings suggest that melatonin treatment blocked the effect of soluble Aβ_1–42_ on Notch1 expression and activation.

**Fig. 6:**
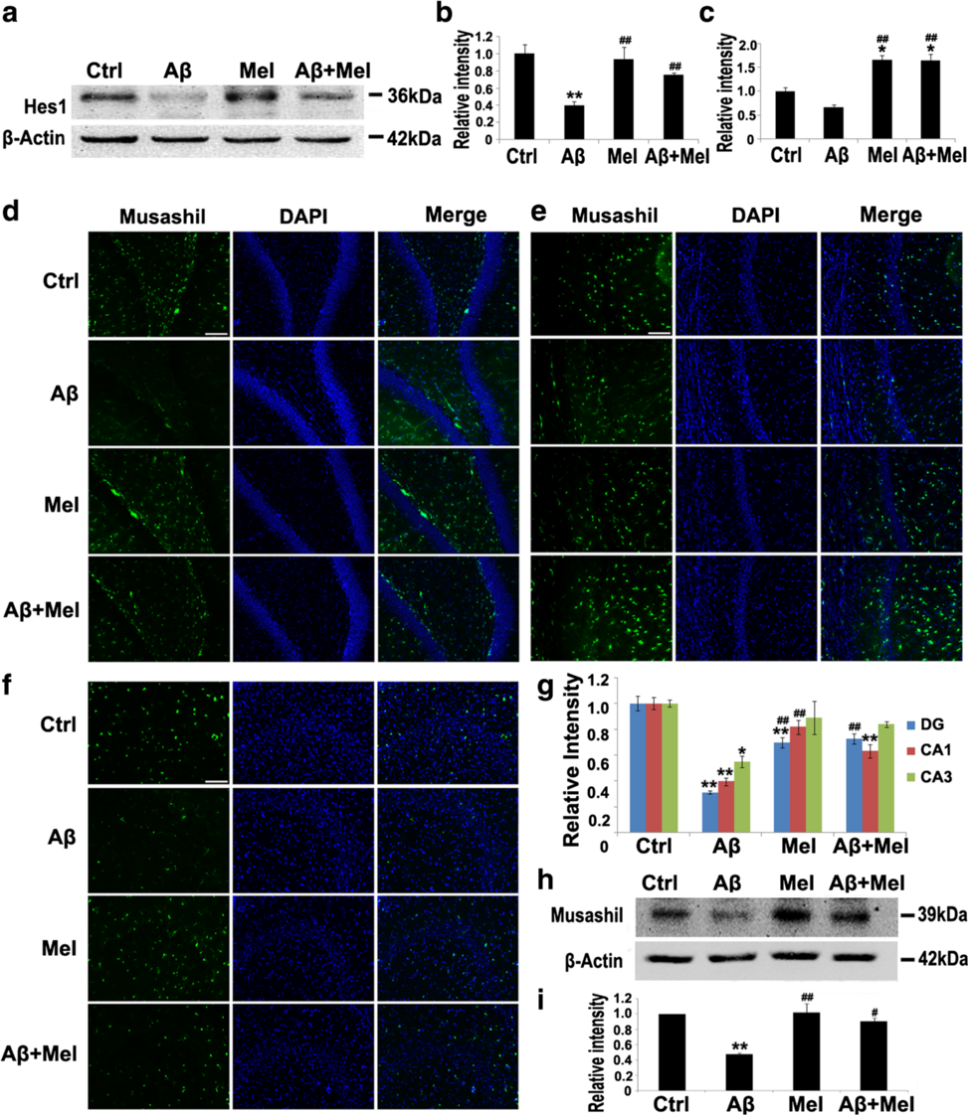
Melatonin increased Hes1 and Musashi1 expression in soluble Aβ_1–42_-injected rats. **a** Representative western blot images for Hes1 in the hippocampus. **b** Statistical analysis of the relative intensity of Hes1 immunoreactive bands. **c** *Hes1* gene expression in the hippocampus analyzed by qPCR. **d** Representative images of immunofluorescent staining for Musashi1 in the dentate gyrus (*DG*) of the hippocampus. **e** Representative images of immunofluorescent staining for Musashi1 in the CA1 region of the hippocampus. **f** Representative images of immunofluorescent staining for Musashi1 in the CA3 region of the hippocampus. **g** Statistical analysis of immunofluorescent staining for Musashi1. **h** Representative western blot images for Musashi1 in the hippocampus. **i** Statistical analysis of the relative intensity of Musashi1 immunoreactive bands. *Scale bar* = 100 μm. *p < 0.05 and **p < 0.01 compared with sham-operated rats; ^#^ *p* < 0.05 and ^##^ *p* < 0.01 compared with Aβ_1–42_-injected rats. *Ctrl* control group, *Aβ* Aβ_1–42_-injected group, *Mel* melatonin-injected group, *Aβ + Mel* dual Aβ_1–42_ and melatonin-injected group, *DAPI* 4′,6-diamidino-2-phenylindole

We investigated whether other components of Notch1 signaling, including Hes1 (a downstream target of Notch1) and Musashi1 (a positive regulator of Notch1 signaling), contributed to the protective effect of melatonin against Aβ_1–42_. We initially examined the effects of melatonin and Aβ on the Hes1 expression using western blot analysis. Compared with the control group, the Hes1 expression was significantly decreased in the Aβ group; however, it was similar to the controls in the Aβ + Mel group (Fig. 6a, b). The *Hes1* gene expression was also decreased in the Aβ group compared with the control group; however, the difference was not significant (Fig. 6c). In the Mel and Aβ + Mel groups, the *Hes1* gene expression was significantly increased compared with the Aβ or control groups (Fig. 6c). We also investigated the Musashi1 expression using immunohistochemistry and western blotting. The Musashi1 expression was significantly decreased in the DG and the CA1 and CA3 regions of the Aβ group compared with the control group, and the reduction was completely inhibited in the DG of the Aβ + Mel group (Fig. 6d–g). Our western blotting results confirmed that the Musashi1 expression was significantly decreased in the hippocampus of the Aβ group compared with the control group, and this reduction was completely prevented in the Aβ + Mel group (Fig. 6h, i). Melatonin treatment alone did not exert a significant effect on the Musashi1 expression (Fig. 6h, i). These findings suggest that melatonin exerts a protective effect against Aβ_1–42_-induced astrogliosis and synaptotoxicity through the regulation of Hes1 and Musashi1 expression.

**Fig. 7:**
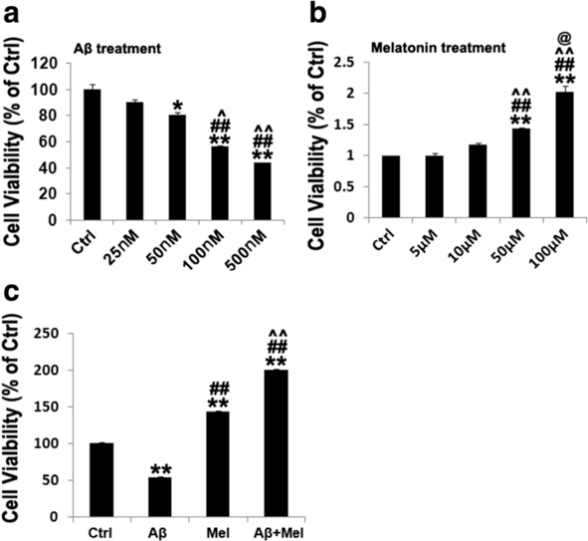
Effects of Aβ_1–42_, melatonin, and Aβ_1–42_ plus melatonin on cell viability in hippocampal neurons. **a** Dose-dependent effect of Aβ_1–42_ on cell viability. *p < 0.05 and **p < 0.01 compared with control cells; ^##^ *p* < 0.01 compared with 25 nM Aβ_1–42_-treated cells; ^^^ *p* < 0.05 and ^^^^ *p* < 0.01 compared with 50 nM Aβ_1–42_-treated cells. **b** Dose-dependent effect of melatonin on cell viability. **p < 0.01 compared with control cells; ^##^ *p* < 0.01 compared with 5 μM melatonin-treated cells; ^^^^ *p* < 0.01 compared with 10 μM melatonin-treated cells; ^@^ *p* < 0.05 compared with 50 μM melatonin-treated cells. **c** Effect of 50 μM melatonin on cell viability of 100 nM Aβ_1–42_-treated hippocampal neurons. **p < 0.01 compared with control cells; ^##^ *p* < 0.01 compared with Aβ_1–42_-treated cells; ^^^^ *p* < 0.01 compared with melatonin-treated cells. *Ctrl* control cells, *Aβ* Aβ_1–42_-treated cells, *Mel* melatonin-treated cells, *Aβ + Mel* Aβ_1–42_ and melatonin-treated cells

To provide more direct evidence for the protective effect of melatonin against Aβ_1–42_-induced neurotoxicity and astrogliosis via the modulation of the Notch1 signaling pathway, in vitro studies were conducted. Neuronal toxicity in primary hippocampal cultures was assessed using a cell viability assay. We initially assessed the dose-dependent effects of a 24-hour Aβ_1–42_ treatment and 26-hour melatonin treatment on neuronal toxicity and neuronal protection, respectively. Neurons exhibited a dose-dependent decrease in the cell viability following Aβ_1–42_ exposure, and 100 nM of Aβ_1–42_ was the lowest dosage to induce a significant reduction in neuronal survival (Fig. 7a). Melatonin exhibited a dose-dependent neuroprotective effect, and 50 μM was the minimum effective dose (Fig. 7b). We further assessed the neuroprotective effect of melatonin (50 μM, 26 hours) against Aβ_1–42_ (100 nM, 24 hours) in primary hippocampal neuronal cultures. Aβ_1–42_ induced a significant reduction in the cell viability compared with the control cells, and the cell viability was substantially increased in the Aβ + Mel-treated neurons compared with the Aβ-treated cells (Fig. 7c). Melatonin treatment alone caused a significant increase in the cell viability compared with the control cells (Fig. 7c).

**Fig. 8:**
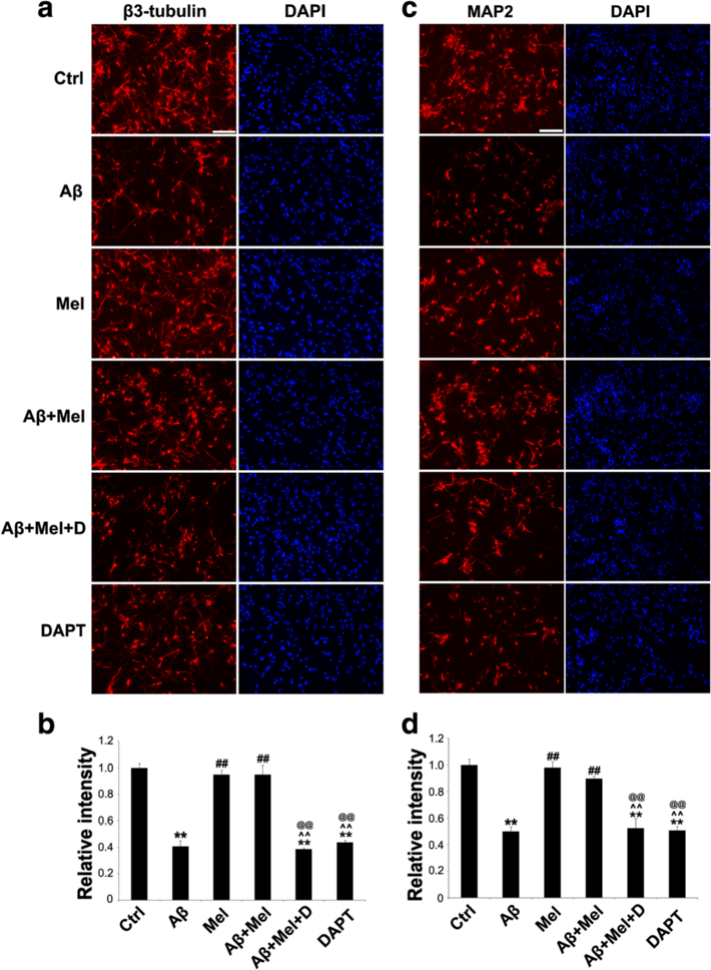
DAPT, an inhibitor of Notch1 signaling, blocked the neuroprotective effects of melatonin against Aβ_1–42_ in hippocampal neurons. **a** Representative images of immunofluorescent staining for βIII-tubulin. **b** Statistical analysis of immunofluorescent staining for βIII-tubulin. **c** Representative images of immunofluorescent staining for MAP2. **d** Statistical analysis of immunofluorescent staining for MAP2. *Scale bar* = 100 μm. **p < 0.01 compared with control cells; ^##^ *p* < 0.01 compared with Aβ_1–42_-treated cells; ^^^^ *p* < 0.01 compared with melatonin-treated cells; ^@@^ *p* < 0.01 compared with Aβ_1–42_ and melatonin-treated cells. *Ctrl* control cells, *Aβ* Aβ_1–42_-treated cells, *Mel* melatonin-treated cells, *Aβ + Mel* Aβ_1–42_ and melatonin-treated cells, *Aβ + Mel + D* Aβ_1–42_, melatonin, and DAPT-treated cells, *DAPT* DAPT (a γ-secretase inhibitor)-treated cells, *DAPI* 4′,6-diamidino-2-phenylindole

To confirm whether the neuroprotective effect of melatonin occurs via Notch1 signaling pathway regulation, we used DAPT, a γ-secretase inhibitor, to inhibit notch1 signaling. Using immunohistochemistry, Aβ_1–42_ significantly decreased the expression of βIII-tubulin (axonal marker) and MAP2 (dendritic marker) in the primary cultured hippocampal neurons compared with the control cells (Fig. 8a–d). The expression of βIII-tubulin and MAP2 was significantly upregulated in the Aβ + Mel-treated cells compared with the Aβ-treated cells (Fig. 8a–d). These findings are consistent with our in vivo findings. However, the βIII-tubulin and MAP2 expression was significantly reduced in the Aβ + Mel + DAPT-treated neurons compared with the Aβ + Mel-treated neurons and was not significantly different compared with the Aβ-treated cells (Fig. 8a–d). The βIII-tubulin and MAP2 expression was also significantly reduced in the DAPT-treated neurons compared with the control group (Fig. 8a–d). These findings suggest that the neuroprotective effect of melatonin against Aβ_1–42_ occurs via the Notch1 signaling pathway.

**Fig. 9:**
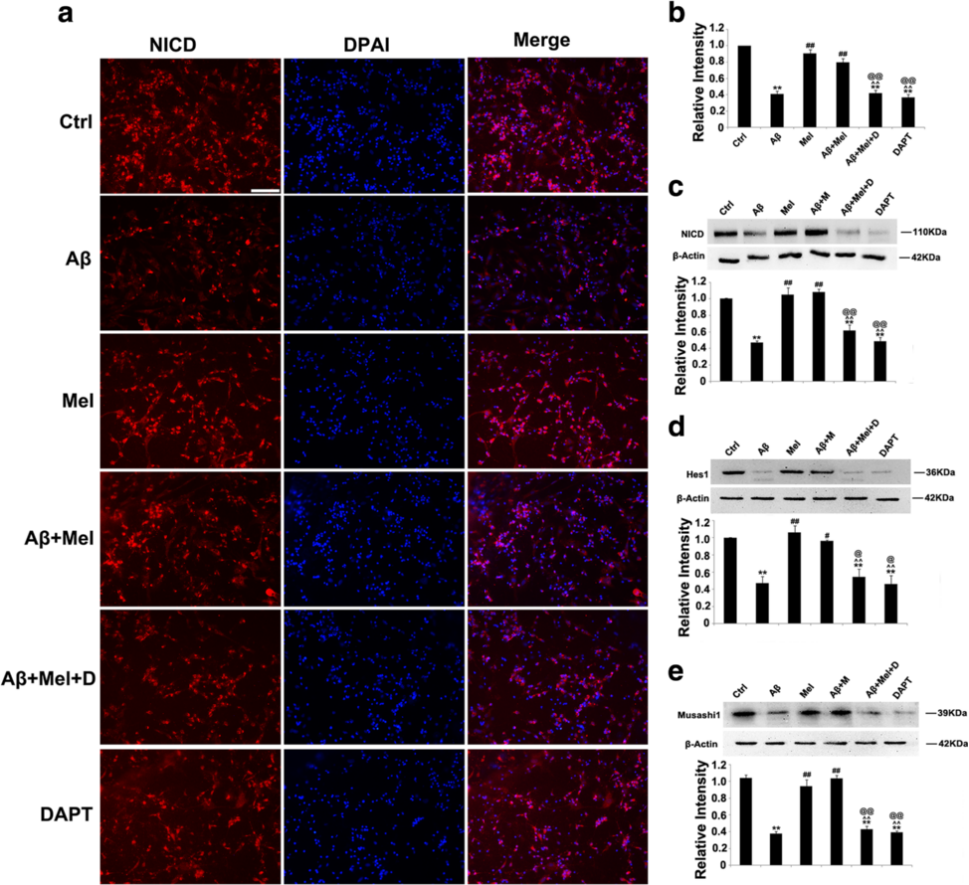
DAPT blocked the effects of melatonin on Notch1, Hes1, and Musashi1 in Aβ_1–42_-treated hippocampal neurons. **a** Representative images of immunofluorescent staining for NICD. **b** Statistical analysis of immunofluorescent staining for NICD. **c** Representative images and statistical analysis of western blots for NICD. **d** Representative images and statistical analysis of western blots for Hes1. **e** Representative images and statistical analysis of western blot for Musashi1. *Scale bar* = 100 μm. **p < 0.01 compared with control cells; ^#^ *p* < 0.05 and ^##^ *p* < 0.01 compared with Aβ_1–42_-treated cells; ^^^^ *p* < 0.01 compared with melatonin-treated cells; ^@@^ *p* < 0.01 compared with Aβ_1–42_ and melatonin-treated cells. *Ctrl* control cells, *Aβ* Aβ_1–42_-treated cells, *Mel* melatonin-treated cells, *Aβ + Mel* Aβ_1–42_ and melatonin-treated cells, *Aβ + Mel + D* Aβ_1–42_, melatonin, and DAPT-treated cells, *DAPT* DAPT (a γ-secretase inhibitor)-treated cells, *DAPI* 4′,6-diamidino-2-phenylindole

We also verified the inhibitory effect of DAPT on the Notch1 signaling pathway. In primary neuronal cultures, immunohistochemistry and western blotting results indicated that the expression of NICD, Hes1, and Musashil1 was substantially decreased in the Aβ + Mel + DAPT-treated cells compared with the Aβ + Mel-treated cells (Fig. 9a–e). These findings indicate that DAPT efficiently inhibited Notch1 signaling pathway activation under our experimental conditions.

## Discussion

Our data indicate that the repeated i.c.v. administration of low doses of soluble Aβ_1–42_ successfully represented some aspects of an in vivo AD model in rats. Melatonin protected against Aβ_1–42_-induced memory deficits, neuroplasticity abnormalities, and astrogliosis under our experimental conditions. Our findings also indicate that Notch1 signaling pathway activation by melatonin may play an important role in its neuroprotection against soluble Aβ_1–42_.

Increasing evidence suggests that soluble Aβ_1–42_ oligomers play a central role in AD pathology [4–7]. Recently, several studies have demonstrated that repeated i.c.v. injections of low doses of Aβ comprised a more effective and reliable method to induce AD-like learning and memory impairments [27, 32, 33]. The use of this method in awake and freely moving animals better resembles AD development compared with a single administration of a large dose of Aβ [27, 32, 33]. In our study, we used this repeated Aβ administration method with modifications to establish an in vivo AD rat model. The repeated i.c.v. injection of soluble Aβ_1–42_ oligomers successfully produced learning and memory impairments, astrogliosis, and synaptic dysfunction in adult rats under our experimental conditions (Figs. 2, 3 and 4). Our study provides additional evidence that the repeated i.c.v. administration of low-dose soluble Aβ_1–42_ oligomers is a simple and reliable approach to develop a rat model with AD-like cognitive impairments, astrocyte activation, and synaptic dysfunction.

Melatonin is a multifunctional neurohormone and has many beneficial effects on AD, including actions as an antioxidant, neuroprotectant, regulator of mitochondrial function, and anti-neuroinflammation agent [14, 34, 35]. Recent studies have indicated that melatonin also attenuated memory deficits, Aβ accumulation, and impairment of neuroplasticity in a sporadic AD rat model [18, 36]. Therefore, melatonin is a potential therapeutic agent for AD prevention and treatment. Our combined in vivo and in vitro studies confirmed that melatonin exerts multiple protective actions against Aβ_1–42_-induced memory deficits, astrogliosis, and impaired neuroplasticity. These observations are consistent with previous studies [18, 36]. In recent studies, the Notch1 signaling pathway was identified as an important signaling pathway for normal adult brain function, as well as in AD and other neurodegenerative diseases [37–39]. The role of Notch1 signaling in the pathogenesis of AD remains controversial; however, there is evidence that the modulation of Notch1 signaling may restore neurogenesis and cognitive functioning in AD animal models [40]. Nevertheless, it remains unknown whether Notch1 signaling is involved in the neuroprotective effects of melatonin in AD and other neurodegenerative diseases. The Notch1/Hes1 signaling pathway participates in the melatonin-mediated cardioprotective effects demonstrated in both in vivo and in vitro models of myocardial ischemia–reperfusion injury [26]. In our study, soluble Aβ_1–42_ suppressed the expression of Notch1, NTMF, and NICD, an active form of Notch1; furthermore, melatonin treatment prevented the Aβ_1–42_-induced reduction of Notch1, NTMF, and NICD in vivo and in vitro. These findings suggest that the Notch1 signaling pathway is involved in the effects of melatonin on soluble Aβ_1–42_-induced memory deficits, impairment of neuroplasticity, and astrogliosis. Additional studies are required to determine whether Notch1 signaling is involved in other melatonin-mediated protective effects in AD and other age-associated neurodegenerative diseases.

The gene and protein expression of Hes1, an essential downstream effector of Notch1 signaling, was consistently regulated by melatonin in our studies, which provides further evidence for the regulatory role of melatonin on Notch1 signaling [11]. Musashi1 comprises an important positive regulator of Notch1 signaling by inhibiting the translation of Numb, an inhibitor of the pathway [12]. Numerous studies have indicated that Numb may play an important role in the pathogenesis and progression of AD [41]; however, the link between Musashi1 and AD remains unclear. Similar to our findings for the Notch1 receptor and NICD, the expression of Hes1 and Musashi1 was decreased by Aβ_1–42_ treatment, and melatonin completely or partially prevented the effects of Aβ_1–42_ on Hes1 and Musashi1. These findings indicate that Hes1 and Musashi1 are involved in the melatonin-mediated protection against Aβ_1–42_. In addition to these two factors, the roles of other Notch signaling components require further investigation.

The inhibition of γ-secretase inhibits the cleavage of the Notch1 receptor by blocking Notch1 signal transduction [8, 10]. DAPT, a commonly used γ-secretase inhibitor, is a powerful inhibitor of Notch1 signaling [8, 10]. We demonstrated that DAPT treatment completely blocked the protective effect of melatonin against Aβ_1–42-_induced neurotoxicity in vitro. We also confirmed that DAPT blocked Notch1 signaling by decreasing NICD, Hes1, and Musashi1 expression. These findings suggest that the protective effect of melatonin against Aβ_1–42_ is dependent on the Musashi1/Notch1/Hes1 signaling pathway. γ-secretase inhibitors also directly interfere with Aβ production; thus, another strategy to block Notch1 signaling will be necessary to better understand the precise connection between Notch1 signaling and the neuroprotective effects of melatonin on AD. Furthermore, Notch1 signaling is dynamically regulated during brain development and disease processes via crosstalk with many signaling pathways. Therefore, additional studies are required to elucidate the precise mechanism of melatonin in Notch1 signaling modulation in AD.

## Conclusions

The current findings demonstrate that the repeated i.c.v. administration of low-dose soluble Aβ_1–42_ oligomers comprises a simple and effective rat model of AD to investigate novel therapeutic approaches for AD and their associated mechanisms. We also demonstrated that the inhibition of Musashi1/Notch1/Hes1 signaling may play an important role in soluble Aβ_1–42_-mediated learning and memory deficits, neuroplasticity dysfunction, and astrogliosis. Furthermore, melatonin may protect against soluble Aβ_1–42_-mediated learning and memory deficits, neuroplasticity dysfunction, and astrogliosis through Musashi1/Notch1/Hes1 signaling pathway activation. The present study suggests that the Musashi1/Notch1/Hes1 signaling pathway plays a key regulatory role in melatonin-mediated effects on AD progression. Moreover, melatonin represents a promising therapeutic agent against AD and other age-associated neurodegenerative diseases.

## Abbreviations

AD: Alzheimer’s disease
ANOVA: Analysis of variance
AraC: Cytosine arabinoside
Aβ: Amyloid-beta
Aβ + Mel: Aβ_1–42_ plus melatonin
Aβ + Mel + D: Aβ_1–42_ plus melatonin and DAPT
AβO: Soluble Aβ oligomer
BCA: Bicinchoninic acid
bHLH: Basic helix–loop–helix
BSA: Bovine serum albumin
cDNA: Complementary DNA
CSF: Cerebrospinal fluid
Ctrl: Control
DAPI: 4′,6-diamidino-2-phenylindole
DAPT: γ-secretase inhibitor
DDW: Double-distilled water
DG: Dental gyrus
E0.5: Embryonic day 0.5
EDTA: Ethylenediaminetetraacetic acid
FAD: Familial AD
FBS: Fetal bovine serum
GAPDH: Glyceraldehyde 3-phosphate dehydrogenase
GFAP: Glial fibrillary acidic protein
HBSS: Hank’s balanced salt solution
HES: Hairy and enhancer of split
Hes1: Hairy and enhancer of split 1
HS: Horse serum
i.c.v.: Intracerebroventricular
i.p.: Intraperitoneal
IgG: Immunoglobulin G
MAP2: Microtubule-associated protein 2
Mel: melatonin
MEM: Minimum Essential Media
MTT: 3-(4,5 –dimethylthiazol–2-yl)-2,5-diphenyltetrazolium bromide
MWM: Morris Water Maze
NDS: Normal donkey serum
NE: Northeast
NICD: Notch1 intracellular domain
NTMF: Notch1 transmembrane fragment
NW: Northwest
PBS: Phosphate-buffered saline
PFA: Paraformaldehyde
PVDF: Polyvinylidene difluoride
qPCR: Quantitative real-time PCR
RIPA: Radio-immunoprecipitation assay
RT: Room temperature
SD: Sprague–Dawley
SE: Southeast
SNAP25: Synaptosomal protein of 25 kDa
SW: Southwest
TBS: PBS containing 0.1 % Triton-X100
TBST: Tris-buffered saline with Tween 20
